# Comparison of Different 3D Surface Registration-Based Methods to Assess Facial Asymmetry

**DOI:** 10.3390/diagnostics14222573

**Published:** 2024-11-15

**Authors:** Annalisa Cappella, Riccardo Solazzo, Luisa Gigante, Alice Gervasoni, Daniele Maria Gibelli, Claudia Dolci, Gianluca Martino Tartaglia, Chiarella Sforza

**Affiliations:** 1 U.O. Laboratory of Applied Morphology, IRCCS Policlinico San Donato, 20097 San Donato Milanese, Italy; 2 Department of Biomedical Sciences for Health, University of Milan, 20133 Milan, Italy; 3 LAFAS (Laboratory of Functional Anatomy of the Stomatognathic System), Department of Biomedical Sciences for Health, University of Milan, 20133 Milan, Italy; 4 Department of Biomedical, Surgical and Dental Sciences, University of Milan, 20122 Milan, Italy; 5 Fondazione IRCCS Cà Granda, Ospedale Maggiore Policlinico, 20122 Milan, Italy

**Keywords:** facial asymmetry, three-dimensional imaging, stereophotogrammetry, 3D facial analysis, Bland–Altman, method comparison

## Abstract

Background/Objectives: Facial asymmetry is gaining an increasing diagnostic interest in many clinical contexts. Several three-dimensional surface-based methods have been proposed for its assessment; however, they might provide non-equivalent data. Since there is a lack of comparative studies in these terms, this study aims to compare three methods for assessing the asymmetry of the face and facial thirds, thus addressing whether the potential differences can be considered clinically acceptable or not. Methods: Two ‘maxillofacial’ methods based on the trigeminal nerve distribution and one ‘orthodontic’ method based on reference horizontal planes were used to identify the facial thirds on 3D facial models of 80 Italian healthy adults to calculate the asymmetry of the face, and the upper, middle, and lower thirds of the face differently selected by each method. As a measure of asymmetry, the Root Mean Square value was calculated through a mirroring surface-based registration. Intra- and inter-operator reliability was verified for each method. Differences and interchangeability between the methods were tested, respectively, by two-way repeated measures ANOVA (Analysis of Variance) and Bland–Altman and Similarity Percentage model analysis. Additionally, the time required to perform each method was assessed. Results: All methods demonstrated excellent intra- and inter-operator reliability. While the ANOVA analysis found significant differences (*p* < 0.001) for the majority of facial Regions of Interest between each method, the Bland–Altman analysis revealed that the differences were clinically acceptable (<0.50 mm) for all facial regions between the trigeminal methods, and for the face and the upper third of the face between the orthodontic method, which was revealed to be faster, and the trigeminal ones. The additional similarity percentage model provided visual support for the complete interchangeability of the two trigeminal methods, as evidenced by the lower Coefficient of Variation value. Conclusions: There is no best method for assessing facial asymmetry that applies to all types of clinical settings, as we have shown that different methods may not be completely interchangeable. However, we suggest that the methods based on the trigeminal subdivision can be used interchangeably in contexts where the morpho-functional analysis of maxillofacial regions with different embryological origins is considered. Thus, the clinical setting imposes the choice of one method over another and, as we have pointed out, the consequent comparison of data with those obtained with methods whose interchangeability has been demonstrated.

## 1. Introduction

The study of facial asymmetry holds significant importance in various fields, ranging from medicine and psychology to aesthetics and anthropology. The human face reflects both genetic and environmental influences [1,2], and its assessment and understanding might provide valuable insights into biological developmental processes [3,4], health and societal conditions and dynamics [5], and even psychological well-being [6,7].

In the biomedical realm, it can be indicative of health issues, such as genetic and/or developmental disorders [8,9,10], hormonal stresses [11], and functional impairments [12]. Its detection might aid in identifying potential health concerns, guiding towards accurate diagnosis and appropriate interventions: its reduction is one of the key goals of aesthetic, plastic, and reconstructive maxillofacial and orthognathic surgical procedures for aesthetic purposes, functional restoration, and the psychological well-being of the patient [13]. Beyond the medical and surgical domains, researchers in psychology and anthropology explored facial asymmetry as a potential marker for attractiveness and mate selection. Several studies suggested that facial symmetry may be linked to the perception of beauty and reproductive fitness, influencing social interactions and mate preferences [14,15], while others found no effects on attractiveness [16,17,18].

Concerning facial morphology, symmetry indicates the correspondence in the shape, size, and arrangement of facial structures on the opposite sides of the median sagittal plane [19]. A slight facial asymmetry level is always present in normal biological situations [10], as soft tissues, bones, and teeth altogether contribute to it. On the contrary, high levels of facial asymmetry might reveal anatomical and functional disturbances between the three components [10,20]. Regardless of the aetiology of facial asymmetry, which can be congenital or acquired [9,21,22,23,24,25,26,27,28], its estimation in healthy populations is crucial for defining an accurate clinical threshold value able to discriminate between normal and pathological asymmetry or asymmetry linked to other conditions [29]. Since perfect symmetry in humans continues to be a theoretical concept [30,31], reconstructive surgical procedures and orthodontic treatments to restore facial harmony or functional symmetry would benefit from accurate diagnoses instead of subjective ones.

The advent of three-dimensional (3D) surface imaging techniques has offered additional diagnostic tools for clinical purposes [32]: they allow us to investigate facial anatomical structures from different perspectives [33] and to quantify various parameters [34]. Facial asymmetry has been widely explored by 3D imaging techniques: parameters like linear, angular, and surface distances from the plane of symmetry [35,36,37,38], asymmetry indexes [36], and asymmetry scores [39] have been thoroughly investigated. However, facial asymmetry analysis may concern the thirds of the face (the so-called ‘facial thirds’) in addition to the entire face, although they may be defined or selected differently by researchers working in different fields and clinical contexts, making asymmetry values reported by diverse studies incomparable.

Clinically significant thresholds of facial asymmetry allowing for the discrimination between what we consider normal from all the other conditions are essential, regardless of the kind of technique used (i.e., two-dimensional (2D) or 3D), the measurements considered, and the clinical context. Through the use of highly reliable and accurate 3D surface-based methods, researchers have described the normal levels of facial asymmetry in populations of different ages, sexes, and geographical origins [40,41,42,43,44,45,46,47,48]. However, data reported in the literature are still not sufficient on their own to define significant thresholds to be commonly used in clinical practices: the ranges of normative values rely on different techniques and different protocols to divide facial areas [29].

Altogether, research on facial asymmetry would largely benefit from the development of reliable and interchangeable methods potentially adopted by the entire scientific community, which, however, seems very improbable. A feasible objective to reach a consensus on reference values of facial asymmetry is to conduct comparative studies testing whether diverse protocols/methods might be considered clinically equivalent and so interchangeable. The purpose of our study is to compare values of facial asymmetry, particularly those of hemifaces and thirds of the face (upper, middle, and lower), examined by mirroring surface registration-based methods differing in the protocol to select the facial thirds (landmarks-dependent or independent Regions of Interest (ROIs)) and envisage, or not, the ROI divisions along the midsagittal plane. Although it is rational to hypothesise differences between the values of asymmetry (expressed in average point-to-point distance calculated as root mean square (RMS)) provided by the three diverse methods compared, our study attempts to address whether the differences should be considered clinically acceptable or not, and so, in the latter case, interchangeable. To reach this objective, the present study tested three different 3D surface registration-based procedures in a large sample of healthy subjects: two mainly used for surgical purposes (both based on trigeminal anthropometric landmarks) and one mostly applied to clinical dentistry (based on reference planes). The agreement was verified through the Bland–Altman analysis and visualised by a percentage similarity model. Overall, this study aims to open a discussion on the beginning of an iterative process to achieve scientific consensus on the diagnosis of facial asymmetry and to promote its application in different clinical settings.

## 2. Materials and Methods

The study sample included 3D facial models of 80 Italian subjects aged between 18 and 95 years old (mean 37.7 ± 16.5 years) of both sexes (37 males; 43 females). The sample included 3D facial models of healthy subjects with no history of craniofacial trauma and surgery, congenital cranio-maxillofacial anomalies and/or dysmorphisms. The 3D facial surface images were captured using the VECTRA^®^ M3 stereophotogrammetric system (Canfield Scientific Inc., Fairfield, NJ, USA), which allows for the scanning of the face in a fast and non-invasive way [49] with high accuracy, reproducibility, and resolution [50]. The image acquisition was performed with the subjects facing the instrument in a rest position with the mouth closed, a neutral expression, and looking straight ahead. A total of 50 anthropometric facial landmarks, according to Ferrario et al. [51], were digitised on the 3D facial models using the manufacturer’s software associated with the instrument, the VECTRA Analysis Module (VAM) (version 5.3.1; Canfield Scientific Inc., Fairfield, NJ, USA).

The retrospective study presented here was approved by the Ethics Committee of the University of Milan (protocol n. 19/24), and it was conducted in accordance with the principles of the Declaration of Helsinki [52]. All the participants included in this study signed an informed consent at the time of enrolment.

### 2.1. Methods for Facial Thirds Selection

Three different methods described in the literature have been used for the selection of the regions of interest (ROIs, facial surfaces), namely, the face/hemiface and the thirds (upper, middle, and lower) of the face, and the assessment of their asymmetry. Two methods refer to the field of cranio–maxillofacial surgery [8,53], while the third one applies to the dentistry field [54]. Regardless of the method applied, the selection of the facial thirds was performed starting from the Facial Areas of Interest (FAIs) selected as described by Codari et al. [53]. The rationale was to standardise the elimination of the confounding areas (e.g., hair, ears, neck) and dispose of corresponding surfaces on which to calculate the asymmetry values with the three methods. All methods described below were applied to all the 3D facial models included in the study sample.

**Figure 1:**
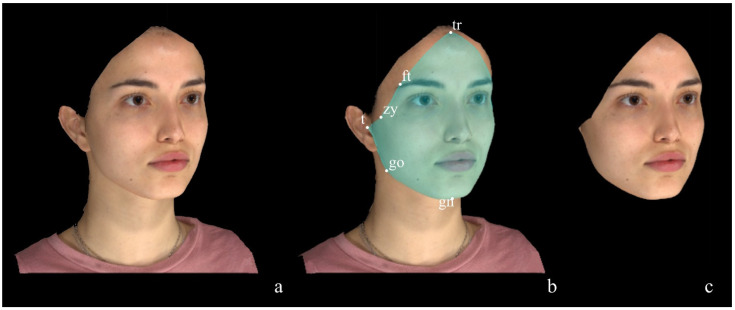
Selection of the facial area of interest. (**a**) 3D model of the participant; (**b**) selection of the FAI through perimetral landmarks; (**c**) FAI.

**Figure 2:**
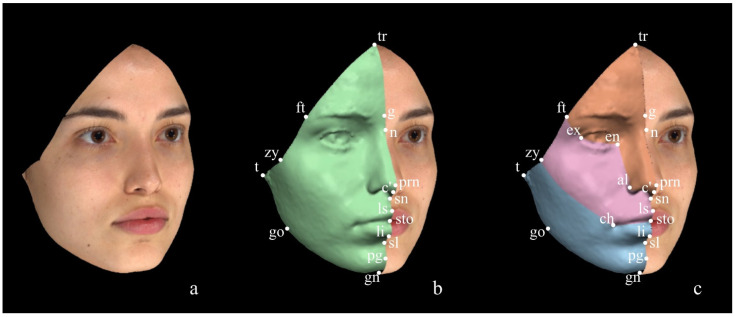
Method 1 for ROI selection. (**a**) FAI; (**b**) hemiface (green); (**c**) thirds of the face: upper third (orange), middle third (pink), lower third (light blue) of the face.

The methods proposed by Codari et al. [53] and Cassi et al. [8] (afterwards named Method 1 and Method 2, respectively) were originally suggested for surgical purposes as they are both based on the territories of trigeminal innervation and, thus, implemented for selecting and analysing the so-called ‘trigeminal thirds’ of the face. Additionally, the method of Codari et al. [53] was also used for forensic purposes [55]. The delimitation of the trigeminal thirds of the face is based on anthropometric landmarks, whose digitisation allows for the standardisation of the selection of the areas of interest, reducing operator dependency. Method 1, proposed by Codari et al. [53], is the reference protocol used in our laboratory and envisages the selection of the Facial Area of Interest (FAI) (the entire facial region to be analysed) using perimetral facial landmarks: trichion (tr), frontotemporale (ft), zygion (zy), tragion (t), gonion (go), and gnathion (gn), as represented in Figure 1. The selected FAI is then divided into the two contralateral hemifaces (right and left), each of which is further subdivided into the trigeminally-defined thirds of the face (upper, middle, lower thirds) to obtain the regions of interest (ROIs) as shown in Figure 2. All anthropometric landmarks used to select the FAI, the hemiface and the thirds of the face are summarised in Table 1.

**Figure 3:**
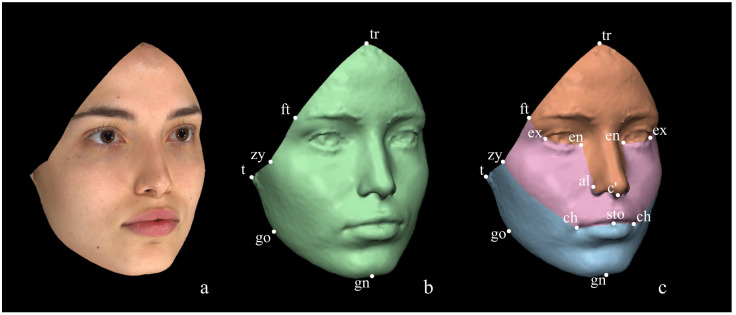
Method 2 for ROI selection. (**a**) FAI; (**b**) face (green); (**c**) facial thirds: upper third (orange), middle third (pink), lower third (light blue) of the face.

Method 2, originally proposed by Cassi et al. [8], although similar to Method 1 [53], differs in several steps. Firstly, the FAI is selected by manually deleting the confounding areas, which allows for the maintenance of a greater amount of surface at the level of the forehead compared to our protocol. Secondly, the FAI is directly divided into the three trigeminal facial thirds without a prior selection of the hemifaces: thus, contrarily to Codari et al. [53], the selected ROIs include both the sides (left and right side together), as depicted in Figure 3. The anthropometric landmarks used by Method 2 for the ROIs selection are listed in Table 2.

**Figure 4:**
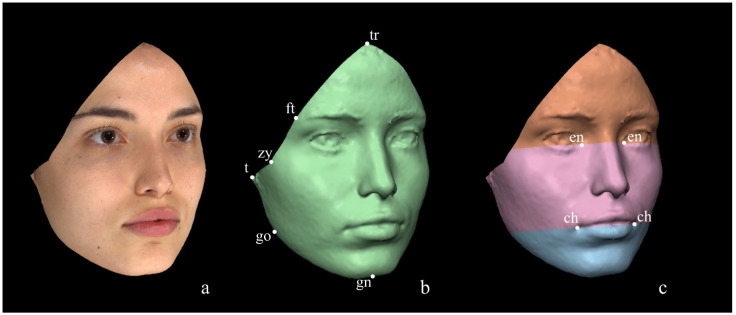
Method 3 for ROI selection. (**a**) FAI; (**b**) face (green); (**c**) facial thirds: upper third (orange), middle third (pink), lower third (light blue) of the face.

Method 3, a protocol mostly used for dentistry purposes, was first described by Primozic et al. [54] and, similarly to Method 2 [8], it manually performs the deletion of the confounding areas and does not laterally divide the facial surfaces. Nonetheless, contrarily to Methods 1 [53] and 2 [8], this method is anthropometric landmarks-semi dependent since it does not exploit the territories of the trigeminal nerve innervation for selecting the facial thirds but rather uses two horizontal planes, each passing for two paired anthropometric landmarks: an upper plane passing through the inner canthi of the eyes (endocanthi) and a lower one passing through the outer commissure of the lips (cheilion) (Figure 4). However, to properly position the horizontal planes, the 3D facial models have to be three-dimensionally oriented in the space according to Mancini et al. [56]: the midsagittal plane is defined from the frontal view of the 3D image as the line (y-axis) passing from the nasion and the pogonion, while the Frankfurt horizontal plane is defined in the lateral view of the 3D image as the line (z-axis) connecting the right ear tragus to the ipsilateral base of the orbit. Once the orientation of the image is achieved, considering the two planes properly positioned, the nasion (n) is set as the origin of the x, y, and z axes having coordinates (0,0,0).

### 2.2. Assessment of the Asymmetry

The asymmetry of all surfaces of interest—faces/hemifaces and facial thirds (upper, middle, and lower thirds of the face)—was assessed for all methods by using the mirroring surface registration-based approach according to previous studies [8,29,40,41,53,57,58,59,60,61]. This approach allows for the superimposing of whichever mirrored ROI, a facial area in our case, onto the corresponding original one, defined as “original” because it is not mirrored. In Method 1 [53], the left side of the ROI is always mirrored and superimposed onto the corresponding contralateral right side (the “original” surface). In Method 2 [8] and 3 [54], the ROIs (FAIs and each third of the face) are first copied and then mirrored and superimposed onto the original ones without a division between the two sides. The registration (superimposition) of the two analysed ROIs is automatically performed by the VAM software (version 5.3.1) through the Iterative Closest Point (ICP) algorithm, which minimises the distance between the two superimposed surfaces by finding their best alignment. The software automatically calculates the point-to-point distance between the two superimposed surfaces, providing a mean distance value in millimetres expressed as Root Mean Square (RMS). Each method was performed on each 3D facial model to obtain a dataset of RMS values for each ROI (face/hemiface and upper, middle, and lower thirds of the face).

In order to verify the reliability of the methods, all protocols were repeated in a sub-sample of 20 individuals by the same operator (L.G.) at two different time-points (at least one month apart) and once by a different operator (A.G.).

Lastly, the time required to apply each of the three protocols, from the selection of the facial thirds to the obtainment of their related RMS, was measured for five randomly selected subjects.

### 2.3. Statistical Analysis

The intra- and inter-operator reliability of the three methods under analysis were assessed through the Intraclass Correlation Coefficient (ICC) and interpreted according to Koo and Li [62].

Descriptive statistics of the RMS values obtained with each method for all the surfaces of interest (face/hemiface and facial thirds) included mean, standard deviation, minimum, and maximum. The normality of the data was evaluated with the Kolmogorov–Smirnov test (α = 0.05), and statistically significant differences between RMS values of the three methods were verified using a two-way repeated measures ANOVA (α = 0.05). In the case of statistically significant differences for the independent variables under analysis (method, facial area, and their interaction), a Bonferroni post-hoc analysis with related correction for multiple comparisons was performed.

The agreement between the methods was specifically verified with the Bland–Altman analysis: mean difference (bias), standard deviation of the bias, 95% Limits of Agreement (LoA), and their 95% confidence interval were calculated. Bland–Altman plots were built to visualise the (dis)agreement between methods and to confirm or exclude their interchangeability. Indeed, the Bland–Altman plot, being an XY scatter plot where the differences of paired measurements (y-axis) are plotted against their mean values (x-axis), allows us to quantify the agreement between two quantitative measurements by calculating the limits of agreement (LoA) [63]. The limits of agreement (LoA) were calculated using the mean difference (bias, $\bar{d}$) and the standard deviation (s) of all differences between paired measurements, and 95% of the differences lie between $\bar{d}$ + 1.96 s and $\bar{d}\;-$ 1.96 s if differences are normally distributed. The normal distribution of the differences was evaluated with a Kolmogorov–Smirnov test (α = 0.05), while the agreement between methods was confirmed for the amplitude of LoA not exceeding 0.50 mm, which is the clinically acceptable value defined accordingly to previous studies [64,65]. Thus, 95% limits of agreement beyond 0.50 mm were deemed clinically unacceptable.

In addition, a percentage similarity model was applied to the dataset of paired measurements of the compared method, according to Scott et al. [66]. The percentage similarity value was calculated with the following formula:

(1) $$\frac{(a+b)/2}{a}\times 100,$$

where *a* is the reference method (Method 1 [53] as the one routinely used by the authors when compared with Method 2 [8] and 3 [54], and Method 2 [8] when compared with Method 3 [54]), and *b* is the method being compared (Method 2 [8] and Method 3 [54]). Percentage similarity histograms were provided to highlight the accuracy and precision of the compared methods, and the agreement between methods was defined by further calculation of the coefficient of variation (CV, calculated by dividing the standard deviation by the mean percentage similarity), allowing for a more meaningful and better visual comparison of methods. According to Scott et al. [66], data pairs with the same value will be 100% similar. Conversely, data pairs in which the comparing method is lesser or greater than the reference one will be lower and greater than 100%, respectively.

Statistical analysis concerning data normality and the construction of the graphs were accomplished using OriginPro (Version 2021b, OriginLab Corporation, Northampton, MA, USA), while all the other analyses were performed with SPSS statistical software (version 29; IBM, Armonk, NY, USA).

## 3. Results

The intra- and inter-observer reliability was evaluated on 3D facial models of 20 subjects randomly selected, and the asymmetry measurements with the three methods were assessed and repeated twice by operator 1 (L.G.) and once by operator 2 (A.G.). The intraclass correlation coefficients (ICCs) for intra-observer repeatability were all greater than 0.962 for all measurements obtained for each facial surface by all three methods. The same was found for the inter-observer reproducibility, for which all the ICCs proved higher than 0.968. According to Koo and Li [62], who suggest excellent reliability for ICC values greater than 0.900, each method demonstrated excellent reliability regardless of the analysed surface, as shown in Table 3.

The mean, standard deviation, minimum, and maximum RMS values of each facial ROI (facial surface) are reported in Table 4 for all methods.

The two-way repeated measures ANOVA detected significant differences for both the independent variables, i.e., the method (*p* < 0.001) and the facial surface (*p* < 0.001), and for their interaction (*p* < 0.001) (Table 5). The partial eta-squared (η_P_^2^) was reported as an estimate of the effect size of the independent variables under analysis and their interaction, and they all showed a large effect when interpreted according to Cohen [67]. Regardless of the facial surface, the posthoc analysis found significant differences in the RMS values of Method 1 [53] and Method 2 [8] and 3 [54] (*p* < 0.001), but not between those of Method 2 [8] when compared with Method 3 [54] (*p* = 0.454). When also considering facial surfaces, the interaction between method and facial surfaces demonstrated significant differences between Method 1 [53] and Method 2 [8] for RMS values of all ROIs, between Method 1 [53] and Method 3 [54] for the RMS values of hemifaces/faces and the middle third of the face, and between Method 2 [8] and Method 3 [54] for RMS values of all the facial thirds (Table 6).

**Figure 5:**
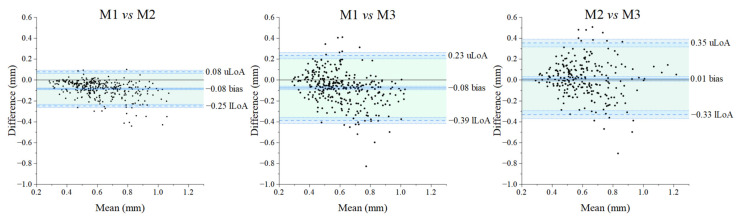
Bland–Altman plots for the overall RMS obtained by the three methods and pairs compared (total data including hemifaces and facial thirds). The black dots represent the differences of paired measurements plotted against their mean values. The continuous blue line indicates the mean difference (bias); superior and lower dashed lines represent the 95% interval of agreement (respectively, upper Limit of Agreement (uLoA) and lower Limit of Agreement (lLoA)). The black continuous line represents the 0. The green area represents the limits of agreement from −1.96*s* to +1.96*s* (*s*: standard deviation of the differences). The light blue areas delimited by dotted blue lines represents the 95% confidence interval of the limits of agreement and bias. The clinical acceptability is set to 0.50 mm, and, whenever the amplitude of the LoA is greater than this value, the methods cannot be considered clinically interchangeable. The plots where Method 1 is compared with Method 2 show clinical acceptability. M: Method.

**Figure 6:**
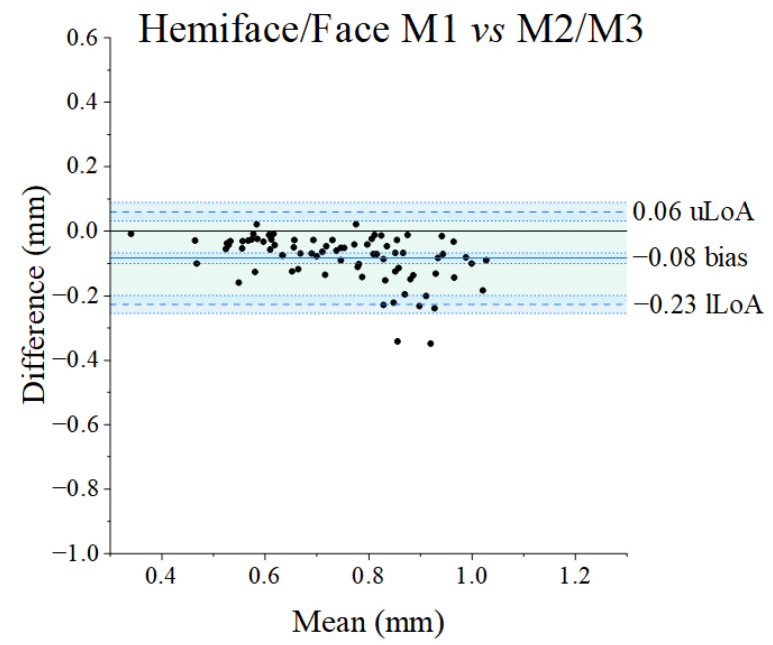
Bland–Altman plots for the RMS of the hemiface obtained by the three methods and pair compared (M2 and M3 are identical). The black dots represent the differences of paired measurements plotted against their mean values. The continuous blue line indicates the mean difference (bias); superior and lower dashed lines represent the 95% interval of agreement (respectively, upper Limit of Agreement (uLoA) and lower Limit of Agreement (lLoA)). The black continuous line represents the 0. The green area represents the limits of agreement from −1.96*s* to +1.96*s* (*s*: standard deviation of the differences). The light blue areas delimited by dotted blue lines represents the 95% confidence interval of the limits of agreement and bias. The clinical acceptability is set to 0.50 mm, and, whenever the amplitude of the LoA is greater than this value, the methods cannot be considered clinically interchangeable. The plots where the hemiface of Method 1 is compared with the face of Methods 2 and 3. M: Method.

**Figure 7:**
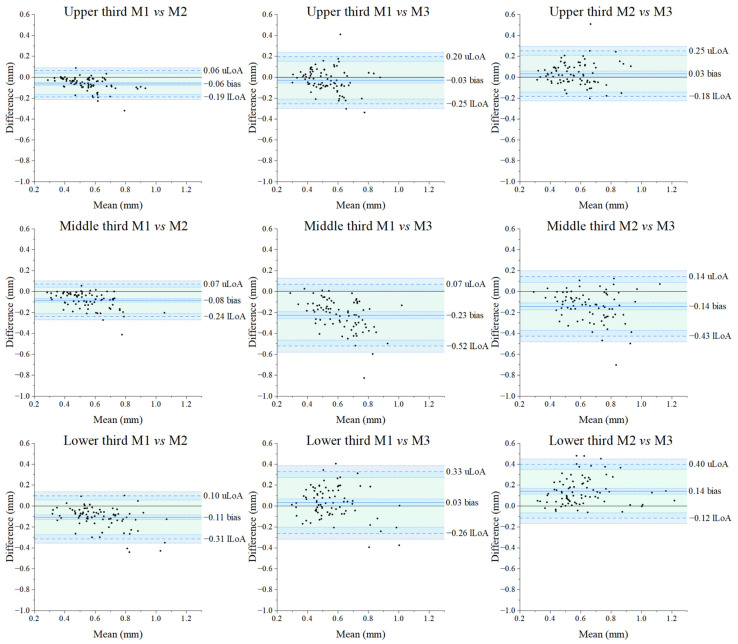
Bland–Altman plots for the RMS of the facial thirds (upper, middle, and lower) were obtained with the three methods and pair compared. The black dots represent the differences of paired measurements plotted against their mean values. The continuous blue line indicates the mean difference (bias); superior and lower dashed lines represent the 95% interval of agreement (respectively, upper Limit of Agreement (uLoA) and lower Limit of Agreement (lLoA)). The black continuous line represents the 0. The green area represents the limits of agreement from −1.96*s* to +1.96*s* (*s*: standard deviation of the differences). The light blue areas delimited by dotted blue lines represents the 95% confidence interval of the limits of agreement and bias. The clinical acceptability is set to 0.50 mm, and, whenever the amplitude of the LoA is greater than this value, the methods are not considered clinically interchangeable. The plots where Method 1 is compared with Method 2 show clinical acceptability for all thirds of the face, with the amplitude < 0.5 mm. Only the plots of the upper third of the face showed clinical acceptability when Method 3 is compared with Method 1 and Method 2. M: Method.

Bland–Altman plots for the paired comparison of the three methods are shown in Figure 5: the x-axes represent the average RMS value for each paired measurement (320 RMS values for each method) across the range of RMS values between 0.25 and 1.25 mm, while the differences between the values of the paired measurements are displayed on the y-axes. The overall mean difference (also called bias) and related standard deviation found between Method 1 [53] and Method 2 [8] and between Method 1 [53] and Method 3 [54] proved very similar (−0.08 ± 0.08 mm and −0.08 ± 0.16 mm, respectively). The bias found between Method 2 [8] and Method 3 [54] proved the lowest (0.01 ± 0.17 mm) although with larger limits of agreements (−0.33 to 0.35 mm, amplitude of 0.68 mm) than those concerning the comparison between Method 1 [53] and Method 2 [8] (amplitude 0.33 mm), but similar to that of Method 1 [53] and Method 3 [54] (amplitude 0.62 mm). The amplitude of Limits of Agreements exceeds the a priori criterion (clinically acceptable) of 0.50 mm in the case of the comparison between Method 1 [53] and Method 3 [54], and Method 2 [8] and Method 3 [54], but not in the comparison between Method 1 [53] and Method 2 [8]. Focusing on data of the specific facial surfaces (Figure 6 and Figure 7), the amplitude of Limits of Agreement (LoA) never exceeds the a priori criterion (0.50 mm) between measurements obtained with Method 1 [53] compared to those of Method 2 [8]. The middle and lower thirds of the face, contrarily to the upper one, exceeded the 0.50 mm clinical threshold for both the comparison of Method 1 [53] and Method 3 [54] and Method 2 [8] and Method 3 [54].

All amplitudes of the limits of agreement are summarised in Table 7.

**Figure 8:**
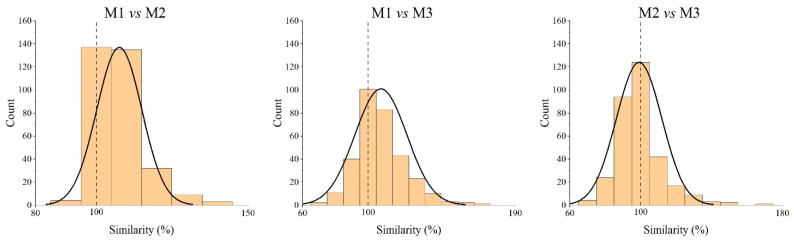
The percentage similarity histogram of each pair method comparison (method 2 and 3 versus method 1 and method 3 versus method 2). The horizontal axis represents the percentage similarity values in intervals, and the vertical axis represents the number (percentage of the total number) of percentage similarity values in each interval.

The percentage similarity model confirmed the results of the Bland–Altman analysis. The percentage similarity values were grouped into intervals and plotted on the horizontal axis (*x*-axis) against the count of values in each interval (*y*-axis), as shown in Figure 8. The mean percentage similarity value was calculated representing the mean bias (expressed as a percentage) of the measurements of one method against those of the other in comparison (the reference, in our case, is Method 1 [53] for the comparison with the other two methods, and Method 2 [8] for the comparison between Method 2 [8] and Method 3 [54]). The closer the mean percentage similarity is to 100%, the closer the mean bias of the two methods will be, which is always expressed in terms of percentage. The Coefficient of Variation (CV), calculated from the mean percentage difference (MDP: mean percentage similarity −100%) and related standard deviation, is reported in Table 8, and the CV assumes a relevant meaning in defining agreement when low. Overall, Methods 2 and 3 provided a similar higher mean bias (7.6% and 7.8%, respectively) when compared to that of our reference method (Method 1, [53]), although Method 2 [8] proved to have better precision (lowest SD) than Method 3 [54] when both were compared with Method 1 [53]. The low CV (6.8%) obtained in the comparison between Method 1 [53] and 2 [8] reflects an agreement with high precision and moderate accuracy. In the comparison between Method 2 [8] and 3 [54], although sharing closer similarity values as demonstrated by the mean percentage similarity, they have a greater dispersion of the data (high SD), and a greater CV value, indicating a poorer agreement.

The time required for the asymmetry assessment applying each of the three protocols of ROI selection to five subjects is reported in Table 9, together with the average time. Method 1 [53] proved to be the one requiring longer times, while Method 3 [54] was the fastest.

## 4. Discussion

The perfectly symmetrical face does not exist in nature as every person, even in normal conditions, presents a certain degree of facial asymmetry [29,44,68,69]. Clinically, facial asymmetry is considered a pathological condition [70], and the search for indices that quantify facial asymmetry, enabling the discrimination between normal biological variability and pathological conditions, is a common goal of many orthodontic, orthognathic, and maxillofacial surgical applications. The success of clinical procedures requires the definition of threshold values to guide treatment decisions, although defining the boundary between facial asymmetry and symmetry is still inherently subjective. The normative data collected in various studies provided insight into where the limit between normality and pathology may lie [8,9,13,29,30,40,41,53,57,59,60,71,72,73], but they are not sufficient on their own to determine the unequivocal clinical threshold. Researchers in the fields of anthropometry, maxillofacial surgery, and dentistry must first develop reliable methods to measure facial asymmetry univocally and, even better, interchangeably: this study represents a first step towards this important goal. The data reported in the literature are often used for comparative purposes in scientific publications and clinical research, but what if these data obtained with different methods do not provide equivalent results? This is an issue of concern in the medical field and is the rationale for the present study.

The interchangeability of the three methods here compared [8,53,54] was verified using Bland–Altman plots [74,75]. All comparisons concerned the asymmetry of the whole face (or hemiface) and specific anatomo–clinical regions of the face (upper, middle, and lower thirds of the face). The Bland–Alman plots show very small values of bias for all comparisons, with ranges of agreement always including the zero value but never in perfect agreement (not equal to zero) [63]. In the comparison between Methods 1 [53] and 2 [8] and Methods 1 [53] and 3 [54], the mean difference between the overall measurements in absolute value is for both comparisons 0.08 mm and 0.01 mm between Methods 2 and 3. Moreover, when considering specific thirds of the face, all the bias values are lower than 0.14 mm, except for the middle third of the face measured with Method 1 [53] when compared to Method 3 [54] (0.23 mm). A further consideration can be drawn on the signs of the bias values: although interchangeable, Method 1 [53] always provides smaller asymmetry values than Method 2 [8]. The impossibility of verifying the real value of facial asymmetry does not allow us to determine whether Method 1 [53] underestimates or Method 2 [8] overestimates facial asymmetry, differently from the trend observed when comparing Methods 1 [53] with Method 3 [54], and Method 2 [8] with Method 3 [54]. However, data suggests that the ‘trigeminal’ methods (Methods 1 [53] and 2 [8]) always provide lower asymmetry values for the middle third of the face and higher values for the lower third of the face when compared to the ‘orthodontic’ method (Method 3 [54]). We speculate that this discrepancy may depend on thirds of the face with different surface extensions that include different anatomical structures. For instance, the middle third of the face assessed by Method 3 [54] includes the nose, a structure that significantly increases the variability of facial asymmetry [76,77,78]. This can explain, in part, the contrasting results in the literature in which the facial third(s) is deemed to be the most symmetric/asymmetric, both in the paediatric [28,29,42,54,79] and the adult population [40,53].

Importantly, Bland–Altman plots are primarily used to assist the clinician in assessing whether or not the differences between the methods being compared are clinically acceptable. Bland and Altman [75] and Mansournia et al. [80] state that the amplitude of the limits of agreement should be used to assess whether the calculated differences are clinically acceptable, as Bland–Altman plots do not provide definitive answers in terms of *p*-values. The acceptable level of agreement between two different techniques or methods is a matter of clinical judgment, and the clinically acceptable limits for bias and limits of agreement or mean error should, therefore, always be defined in advance, depending on the target populations in which the methods/techniques are intended to be used. In our case, a difference of less than 0.50 mm can be considered acceptable and clinically irrelevant, according to some authors [64,65]. Considering this value, although the two-way repeated measures ANOVA suggested the existence of significant differences between the three methods, the analysis cannot confirm if the differences lay under the clinical significance attested to 0.50 mm in the literature [64,65]. The Bland–Altman and the percentage similarity model analysis helped the interpretation of our results: we can propose Methods 1 [53] and 2 [8] as interchangeable methods for any type of facial surface, while Method 3 [54] is equivalent to the other two methods only for the face/hemiface and the upper third of the face, although the amplitude was low (always less than 0.60 mm). This was also confirmed by the percentage similarity model that showed a low coefficient of variation (CV) only between Methods 1 [53] and 2 [8], implying that the interchangeability is clinically acceptable only among the two ‘trigeminal’ methods and not between the ‘trigeminal’ methods with the ‘orthodontic’ one.

Some considerations are due to the execution time of one method over another. When carried out in studies including large population samples, all methods are certainly time-consuming: Method 1 [53] proved to be five times longer than Method 3 [54]. The choice of which method to use would depend on the purposes of application and not on time execution: if the assessment of the middle and lower thirds of the face requires us to capture surgical aspects, then Method 2 [8] has the advantage of being faster. In the case of the clinical evaluation of the asymmetry of the whole face or the upper third of the face, the application of Method 3 [54] could instead represent the elective clinical choice, whatever the nature or the diagnostic purpose of the study is, as it has the advantage of greatly reducing the time.

To the best of our knowledge, this is the first study investigating the (dis)agreement between different 3D surface-based methods. Previous studies have highlighted the difficulties in comparing their own data with those available in the literature because of the different 3D methodologies and statistical approaches used [29], stressing the need to investigate such topics. Cassi et al. [8] pointed out that many authors [40,42,43,48,79] had previously used similar surface registration-based protocols for a global and a local evaluation of the surface discrepancy. However, those approaches differed in the division of the facial areas, as they entailed a horizontal selection of the thirds of the face instead of exploiting the territories of trigeminal innervation defined by anthropometric landmarks, as proposed by Codari et al. [53]. Cassi et al. [8] clearly stated all the differences between their method and the original one: it performs a trigeminal subdivision without demarcation of the midsagittal plane (to reduce the number of anthropometric landmarks to be selected) as well as a registration step avoiding the mid-sagittal subdivision according to other authors [81,82,83] as the ‘individuation’ of the midsagittal plane is a possible misleading factor for the precision of the asymmetry measurement [81]. Although the authors [8] recognised differences between the various 3D methods of ROI selection, they did not investigate possible differences with their proposed method. Our study tried to fill this literature gap by recognising that data on facial asymmetry, when provided by different 3D surface-based methods differing in the selection of the facial surfaces, should not be directly used as comparative data unless their equivalence has been proven in methodological studies such as ours, as this might hinder and mislead the correct clinical and diagnostic interpretation.

The application of advanced 3D optical technologies and equipment in the morphological analysis of the craniofacial district has significantly enhanced the diagnostic process and treatment plan strategy in numerous fields, including orthodontics, anthropometry, maxillofacial surgery, and rehabilitation [34]. In recent years, the use of these technologies has become a pivotal component of both routine clinical practice and cutting-edge research. The detection of craniofacial morphometrical parameters, such as facial asymmetry or craniofacial dysmorphic characteristics, are useful diagnostic markers of support in the characterisation of syndromes or specific pathological conditions. However, to properly apply such technologies and to advance in the assessment of craniofacial morphology, further research efforts are needed to build common and interchangeable protocols/methods to ensure scientific rigour and consensus in the clinical and diagnostic practices in which these are involved.

### Limitations

Although this study provides valuable insights into the methods used to assess facial asymmetry in different clinical settings, there are some limitations that must be addressed as they may impact the generalisability of our results. The limitations are mainly referable to the characteristics of the participants included in this study: we considered only healthy Italian adults. The inclusion of diverse demographic groups could help to validate these methods more broadly and ensure their applicability across diverse populations in terms of geographical origin, health status, and age. Particularly, age is one of the factors impacting facial asymmetry most; since our study focused on a broad age range, this should be considered an additional limitation as a correlation between asymmetry and age has been reported [72,84]. In addition, the same study conducted in non-healthy individuals, particularly those suffering from certain facial pathologies, would be of paramount importance to clinical practices such as maxillofacial surgery or orthodontics to verify the practical applicability and generalizability of these methods.

## 5. Conclusions

We conclude that there is not one single best method to assess facial asymmetry that applies to all types of clinical settings because the diverse methods compared proved to not be completely interchangeable. However, we can suggest that methods based on trigeminal subdivision (Methods 1 proposed by Codari et al. [53] and Method 2 proposed by Cassi et al. [8]) be interchangeably used in maxillofacial settings or in contexts where to consider the morphometric and functional analysis of facial regions with different embryological origins. Contrarily, the orthodontic field should continue to use Method 3 (proposed by Primozic et al. [54]), which represents the elective method for the dentistry field.

The lack of complete interchangeability between the three analysed methods in the assessment of asymmetry recalls the attention of researchers on the use of data previously published. The data should be directly compared only to those obtained with the same method or interchangeable methods.

Because of the relevance of the topic of asymmetry quantification, we also auspicate further steps: (i) future comparative studies for further methods, and (ii) the definition of common threshold values to discriminate the ‘pathological asymmetry’, irrespectively of the method employed.

## Figures and Tables

**Table 1:** Landmarks used for the selection of the facial surfaces in Method 1.

Facial Surface	Landmarks Used for Selection
FAI	trichion (tr), frontotemporale (ft), zygion (zy), tragion (t), gonion (go), gnathion (gn)
Hemiface	trichion (tr), frontotemporale (ft), zygion (zy), tragion (t), gonion (go), gnathion (gn), pogonion (pg), sublabiale (sl), labiale inferius (li), stomion (sto), labiale superius (ls), subnasale (sn), columella (c’), pronasale (prn), nasion (n), glabella (g)
Upper Third	trichion (tr), frontotemporale (ft), exocanthion (ex), endocanthion (en), alare (al), columella (c’), pronasale (prn), nasion (n), glabella (g)
Middle Third	frontotemporale (ft), zygion (zy), cheilion (ch), stomion (sto), labiale superius (ls), subnasale (sn), columella (c’), alare (al), endocanthion (en), exocanthion (ex)
Lower Third	zygion (zy), tragion (t), gonion (go), gnathion (gn), pogonion (pg), sublabiale (sl), labiale inferius (li), stomion (sto), cheilion (ch)

**Table 2:** Landmarks used for the selection of the facial surfaces/thirds of the face in Method 2.

Facial Surface	Landmarks Used for Selection
FAI/Face	trichion (tr), frontotemporale (ft), zygion (zy), tragion (t), gonion (go), gnathion (gn)
Upper Third	trichion (tr), frontotemporale (ft), exocanthion (ex), endocanthion (en), alare (al), columella (c’)
Middle Third	columella (c’), alare (al), endocanthion (en), exocanthion (ex), frontotemporale (ft), zygion (zy), cheilion (ch), stomion (sto)
Lower Third	stomion (sto), cheilion (ch), zygion (zy), tragion (t), gonion (go), gnathion (gn)

**Table 3:** Intra- and inter-operator reliability of the three methods for each facial area of interest and third of the face (upper, middle, and lower thirds).

Method	Facial Area	Intra-Operator Repeatability		Inter-Operator Reproducibility	
		ICC	95% CI	ICC	95% CI
Method 1 [53]	Total	0.982	0.963–0.994	0.991	0.987–0.995
	Hemiface	0.985	0.944–0.991	0.997	0.992–0.999
	Upper Third	0.977	0.944–0.991	0.995	0.987–0.998
	Middle Third	0.962	0.908–0.985	0.968	0.923–0.987
	Lower Third	0.962	0.907–0.985	0.980	0.951–0.992
Method 2 [8]	Total	0.989	0.983–0.993	0.995	0.992–0.997
	Face	0.996	0.991–0.999	0.998	0.994–0.999
	Upper Third	0.970	0.925–0.988	0.995	0.988–0.998
	Middle Third	0.988	0.970–0.995	0.989	0.974–0.996
	Lower Third	0.987	0.969–0.995	0.992	0.971–0.997
Method 3 [54]	Total	0.996	0.993–0.997	0.989	0.983–0.993
	Face	0.996	0.991–0.999	0.998	0.994–0.999
	Upper Third	0.998	0.995–0.999	0.984	0.959–0.994
	Middle Third	0.997	0.993–0.999	0.973	0.935–0.989
	Lower Third	0.985	0.964–0.994	0.990	0.974–0.996

ICC: Intraclass correlation coefficient; CI: Confidence Interval.

**Table 4:** Descriptive statistic data of RMS values (mm) of hemiface/face, upper third, middle third, and lower third of the face calculated by each method.

Method	Facial Surface	Mean (mm)	SD (mm)	Minimum (mm)	Maximum (mm)
Method 1 [53]	Hemiface	0.70	0.14	0.34	0.98
	Upper Third	0.51	0.12	0.27	0.88
	Middle Third	0.50	0.13	0.28	0.95
	Lower Third	0.57	0.15	0.28	1.01
Method 2 [8]	Face	0.79	0.17	0.34	1.11
	Upper Third	0.58	0.15	0.30	0.98
	Middle Third	0.59	0.16	0.29	1.16
	Lower Third	0.68	0.19	0.34	1.24
Method 3 [54]	Face	0.79	0.17	0.34	1.11
	Upper Third	0.54	0.14	0.29	0.94
	Middle Third	0.73	0.19	0.29	1.18
	Lower Third	0.54	0.18	0.29	1.19

SD: Standard Deviation; RMS: Root Mean Square.

**Table 5:** Two-way repeated measures ANOVA results.

Variable	F-Value	*p*-Value	η_P_^2^
Method	26.99	<0.001 *	0.570
Facial surface	80.32	<0.001 *	0.421
Method × Facial Surface	13.66	<0.001 *	0.440

* indicates significant difference at α = 0.05.

**Table 6:** Post-hoc comparisons for the independent variables and their interaction for each facial area of interest and third of the face.

Method	Facial Surface	*p*-Value
Method 1 vs. Method 2		<0.001 *
Method 1 vs. Method 3	All surfaces	<0.001 *
Method 2 vs. Method 3		0.454
Method 1 vs. Method 2	Hemiface/Face	<0.001 *
Method 1 vs. Method 3		<0.001 *
Method 2 vs. Method 3		/
Method 1 vs. Method 2	Upper third of the face	<0.001 *
Method 1 vs. Method 3		0.078
Method 2 vs. Method 3		0.024 *
Method 1 vs. Method 2	Middle third of the face	<0.001 *
Method 1 vs. Method 3		<0.001 *
Method 2 vs. Method 3		<0.001 *
Method 1 vs. Method 2	Lower third of the face	<0.001 *
Method 1 vs. Method 3		0.137
Method 2 vs. Method 3		<0.001 *

* indicates significant difference at α = 0.05.

**Table 7:** Amplitude of limits of agreement in each method comparison for all facial areas of interest, including upper, middle, and lower thirds of the face.

	Amplitude of Limits of Agreements (mm)				
	All Surfaces	Hemiface/Face	Upper Third	Middle Third	Lower Third
Method 1 vs. Method 2	0.33	0.29	0.25	0.31	0.41
Method 1 vs. Method 3	0.62	0.29	0.45	0.59	0.59
Method 2 vs. Method 3	0.68	/	0.43	0.57	0.52

**Table 8:** Summary of the percentage similarity histogram and normal curve statistics.

Method Pair Comparison	Mean Similarity	MPD ± SD	CV
Method 1 * vs. Method 2	107.6%	7.6% ± 7.4%	6.8%
Method 1 * vs. Method 3	107.8%	7.8% ± 15.9%	14.7%
Method 2 * vs. Method 3	99.1%	0.9% ± 12.8%	13.0%

* indicates the reference method within the pair method comparison used as coefficient a in the formula (n = 320); MPD: mean percentage difference; SD: Standard Deviation; CV: Coefficient of variation.

**Table 9:** Time for the application of the three methods.

Subject	Method 1 [53]	Method 2 [8]	Method 3 [54]
1	8′ 40″	5′ 14″	1′ 50″
2	9′ 58″	4′ 43″	1′ 47″
3	11′ 16″	4′ 32″	1′ 43″
4	10′ 07″	4′ 34″	1′ 50″
5	8′ 54″	4′ 37″	1′ 55″
Average	9′ 47″	4′ 44″	1′ 47″

## Data Availability

The data presented in this study are available upon reasonable request from the corresponding author.
